# A conditional model predicting the 10-year annual extra mortality risk compared to the general population: a large population-based study in Dutch breast cancer patients

**DOI:** 10.1371/journal.pone.0210887

**Published:** 2019-01-24

**Authors:** Marissa C. van Maaren, Robert F. Kneepkens, Joke Verbaan, Peter C. Huijgens, Valery E. P. P. Lemmens, Rob H. A. Verhoeven, Sabine Siesling

**Affiliations:** 1 Department of Research, Netherlands Comprehensive Cancer Organisation, Utrecht, the Netherlands; 2 Department of Health Technology and Services Research, Faculty of Behavioural, Management and Social Sciences, Technical Medical Centre, University of Twente, Enschede, the Netherlands; 3 Achmea Pension & Life, Tilburg, the Netherlands; 4 De Hoop Life Reinsurance, the Hague, the Netherlands; 5 Department of Public Health, Erasmus MC University Medical Centre, Rotterdam, The Netherlands; University of South Alabama Mitchell Cancer Institute, UNITED STATES

## Abstract

**Objective:**

Many cancer survivors are facing difficulties in getting a life insurance; raised premiums and declinatures are common. We generated a prediction model estimating the conditional extra mortality risk of breast cancer patients in the Netherlands. This model can be used by life insurers to accurately estimate the additional risk of an individual patient, conditional on the years survived.

**Methodology:**

All women diagnosed with stage I-III breast cancer in 2005–2006, treated with surgery, were selected from the Netherlands Cancer Registry. For all stages separately, multivariable logistic regression was used to estimate annual mortality risks, conditional on the years survived, until 10 years after diagnosis, resulting in 30 models. The conditional extra mortality risk was calculated by subtracting mortality rates of the general Dutch population from the patient mortality rates, matched by age, gender and year. The final model was internally and externally validated, and tested by life insurers.

**Results:**

We included 23,234 patients: 10,101 stage I, 9,868 stage II and 3,265 stage III. The final models included age, tumor stage, nodal stage, lateralization, location within the breast, grade, multifocality, hormonal receptor status, HER2 status, type of surgery, axillary lymph node dissection, radiotherapy, (neo)adjuvant systemic therapy and targeted therapy. All models showed good calibration and discrimination. Testing of the model by life insurers showed that insurability using the newly-developed model increased with 13%, ranging from 0%-24% among subgroups.

**Conclusion:**

The final model provides accurate conditional extra mortality risks of breast cancer patients, which can be used by life insurers to make more reliable calculations. The model is expected to increase breast cancer patients’ insurability and transparency among life insurers.

## Introduction

Cancer incidence is rising, while mortality rates are decreasing[1,2]. The growing number of cancer survivors aims to reintegrate into society, but encounters several problems. While several studies focus on return to work[3–6], another major problem is the difficulty of getting a life insurance, often needed for a mortgage, which is getting far less attention. A study on socio-economic implications of cancer survivorship in the Netherlands, published in 2012, showed that 60% of the applications for a life insurance by cancer survivors was declined[7]. In the Netherlands, life insurers work with internationally available data on cancer survival to estimate a patient’s mortality risk. These data can be different between insurance companies and are not transparent due to life insurers’ competition policy. Providing life insurers identical data to base their decision on may enhance transparency. Besides, Dutch life insurers may make better informed decisions by using data specifically addressing mortality risks for cancer patients and survivors in the Netherlands.

Usually, survival is estimated from diagnosis, which is of limited value for cancer patients who survived several years, since these estimates will be heavily influenced by patients who died during the first years following diagnosis. It is more appropriate to have survival estimates of patients who already survived the years that are relevant for the particular cancer patient[8,9]. By estimating this so-called conditional survival, survival estimates are modified by only including patients still alive at a relevant time point. As breast cancer is one of the most commonly diagnosed cancers in the world[10], this cancer type was selected to study conditional survival in the Netherlands aiming to provide accurate data for life insurers. Conditional survival has been reported for many cancers, among others in breast cancer[8,11–16]. However, prediction models estimating the conditional extra mortality risk, compared to the general population, have not been generated yet. These types of models would not only provide life insurers a better basis for their decision, it also provides medical experts a more objective basis to deem a patient cured of disease[17].

This study aimed to generate a prediction model for breast cancer patients estimating the annual extra mortality risk compared to the general population, conditional on zero to nine years survived.

## Materials and methods

### Study population

We included all female operated patients diagnosed with stage I-III breast cancer in 2005–2006 from the Netherlands Cancer Registry (NCR). This population-based registry contains prospectively registered data on all patients newly diagnosed with cancer from 1989 onwards. The NCR has specialized trained and dedicated registrars who derive patient-, tumor-, and treatment-related characteristics from hospital records of all patients diagnosed with cancer. This study was approved by the privacy committee (Commissie van Toezicht) of the NCR.

### Data collection

Data on patient-, tumor-, and treatment-related characteristics were obtained from the NCR. Tumor topography and morphology were coded according to the International Classification of Diseases for Oncology[18]. Staging was coded using the tumor, node, and metastasis classification system of the International Union Against Cancer, 6^th^ edition[19]. Additional data on vital status and date of death were derived from the Municipal Personal Records database and were complete until February 2017.

### Outcomes

The annual extra risks on mortality until 10 years after diagnosis, using conditional survival points from zero to nine years. The extra risk was defined as the additional risk of a breast cancer patient at a specific time point after diagnosis, compared to the general population, calculated for stage I, II and III separately.

### Statistical analysis

Patient-, tumor-, and treatment-related characteristics were summarized, separated by stage of disease. Annual mortality risks, for every stage of disease, were calculated using conditional logistic regression. This method was chosen as the outcomes of logistic regression can directly be interpreted by life insurers as extra mortality risks per year, which can subsequently be translated into a premium. Conditional risks was estimated by excluding patients who had died at start of every analysis (x years after diagnosis). Extra risks were calculated by subtracting the risk of the general population by the risk of the patient population, where matching took place on age, gender and calendar year. To match the patient population to the general population, we calculated yearly risks (so they could be matched on calendar year). Accordingly, for each stage of disease 10 models were generated calculating the 1-year mortality risk at diagnosis, and one to nine years after diagnosis. Altogether, 30 models were generated. For each stage separately, prognostic variables were included in the model when they significantly contributed to one of the models (p<0.1). Prognostic variables could differ per stage of disease. Furthermore, as occasionally the number of events was limited, some variables were reduced to less categories. Variables included for analyses were: age, tumor stage, nodal stage, lateralization, location within the breast, tumor differentiation grade, histological tumor type, multifocality, hormonal receptor status (ER/PR status), HER2 status, type of surgery, axillary lymph node dissection, use of adjuvant systemic therapy, primary systemic therapy, targeted therapy and radiotherapy. For all patients the pathological tumor and nodal stages were used, except for patients treated with primary systemic therapy, for who the clinical tumor and nodal stages were used. In case of a re-excision, we used the most extensive operation as type of surgery. No stepwise, backward or forward selection was performed, since these methods are shown to result in unstable and unreproducible models and the selected variables are sensitive to random fluctuations in the data[20]. Variance-covariance matrices were used to calculate 95% confidence intervals (CI).

### Validation

Goodness-of-fit of each underlying model was determined by the Hosmer-Lemeshow test in deciles based on the predicted risk. A non-significant test implied that the observed mortality did not differ significantly from the predicted mortality risk[21]. Discriminatory accuracy was determined by the Area Under the Receiver Operating Characteristic (ROC) curve (AUC). An AUC of 0.5 indicates that the model is as good as flipping a coin, while an AUC of 1.0 represents perfect discriminatory accuracy.

For every underlying model, internal validation was performed by manual bootstrapping with 1000 replicates. The model was applied to every bootstrap sample, whereafter the AUC was calculated. The difference between the original and the mean AUC of the 1000 replicates was used as correction factor and subtracted from the original AUC. This bias-corrected AUC was used as a measure for internal validation.

External validation was performed on patients diagnosed in 2007–2008, with the same inclusion criteria as the development population. Only the first eight models of every stage were externally validated, as follow-up for these patients was only completed until eight years from diagnosis.

### Model testing

Before the final model was made available for life insurers, the model was tested for its effect on daily practice. This included the analysis of 50 patients per life insurer with specific characteristics and number of years survived, based on randomly selected records from the NCR (anonymized). In the Netherlands, there are 10 insurance companies specialized in life insurances, which were all invited. Life insurers were asked to provide the mortality risks of the patients based on the currently used guidelines (which differed among life insurers and were confidential). These mortality risks were consequently translated into acceptation (with or without premium raise) or rejection. All results were collected by the Centre for Insurance Statistics in the Netherlands, to comply with the Antitrust legislation, and subsequently anonymously provided to the Netherlands Comprehensive Cancer Organisation (IKNL). For every stage of disease and age category, results of the newly-developed model were compared with results of the currently used guidelines. Specification of the exact levels of premium raise was not possible according to the Antitrust legislation. All statistical analyses were performed in Stata/SE version 14.1 (StataCorp LP) and Microsoft Excel 2016.

## Results

### Study population

The study population comprised 23,234 patients. Patient-, tumor-, and treatment-related characteristics per stage were summarized in Table 1. The mean age was 59 years (interquartile range 49–69 years). Most patients were diagnosed with stage I disease (43.5%) (Table 1). Median follow-up from date of diagnosis to date of last observation was 10.6 years (interquartile range 8.7 to 11.3 years).

**Table 1:** Patient-, tumor-, and treatment-related characteristics.

Characteristics	Stage I (n = 10,101)	Stage II (n = 9,868)	Stage III (n = 3,265)
**Age (years)**			
<40	405 (4.0)	657 (6.7)	261 (8.0)
40–64	5,978 (59.2)	5,804 (58.8)	1,959 (60.0)
≥65	3,718 (36.8)	3,407 (34.5)	1,045 (32.0)
**Pathological tumor stage**			
T1	10,051 (99.5)	3,037 (30.8)	666 (20.4)
T2	-	6,516 (66.0)	1,351 (41.4)
T3	-	273 (2.8)	646 (19.8)
T4	-	-	569 (17.4)
Unknown	50 (0.5)	42 (0.4)	33 (1.0)
**Pathological nodal stage**			
N0	9,835 (97.4)	3,960 (40.1)	154 (4.7)
N1	-	5,761 (58.4)	613 (18.8)
N2	-	-	1,571 (48.1)
N3	-	-	886 (27.1)
Unknown	266 (2.6)	147 (1.5)	41 (1.3)
**Location**			
Outer quadrants	4,740 (46.9)	4,702 (47.7)	1,420 (43.5)
Inner quadrants	2,221 (22.0)	1,724 (17.5)	379 (11.6)
Central parts	623 (6.2)	751 (7.6)	313 (9.6)
Overlapping lesions	2,296 (22.7)	2,517 (25.5)	1,068 (2.6)
Unknown	221 (2.2)	174 (1.8)	85 (2.6)
**Differentiation grade**			
Well	2,979 (29.5)	1,558 (15.8)	289 (8.9)
Moderate	4,424 (43.8)	4,206 (42.6)	1,132 (34.7)
Poor	2,109 (20.9)	3,431 (34.8)	1,399 (42.9)
Unknown	589 (5.8)	673 (6.8)	445 (13.6)
**Histological tumor type**			
Ductal	8,231 (81.5)	7,857 (79.6)	2,523 (77.3)
Lobular	896 (8.9)	1,214 (12.3)	516 (15.8)
Mixed	408 (4.0)	393 (4.0)	163 (5.0)
Other	566 (5.6)	404 (4.1)	63 (1.9)
**Multifocality**			
No	8,528 (84.4)	8,069 (81.8)	2,370 (72.6)
Yes	1,255 (12.4)	1,554 (15.8)	756 (23.2)
Unknown	318 (3.2)	245 (2.5)	139 (4.3)
**Hormonal receptor status**			
ER and PR positive	6,887 (68.2)	6,331 (64.2)	1,811 (55.5)
ER or PR positive	1,631 (16.2)	1,581 (16.0)	605 (18.5)
ER negative	1,286 (12.7)	1,888 (19.1)	837 (25.6)
Unknown	297 (2.9)	68 (0.7)	12 (0.4)
**HER2 status**			
Negative	7,464 (73.9)	7,444 (75.4)	2,281 (69.9)
Unclear	938 (9.3)	354 (3.6)	85 (2.6)
Positive	950 (9.4)	1,457 (14.8)	682 (20.9)
Unknown	749 (7.4)	613 (6.2)	217 (6.7)
**Type of surgery**			
Breast-conserving surgery	6,956 (68.9)	4,670 (47.3)	731 (22.4)
Mastectomy	3,137 (31.1)	5,194 (52.6)	2,533 (77.6)
Unknown	8 (0.1)	4 (0.0)	1 (0.0)
**Axillary lymph node dissection**			
No	8,544 (84.6)	3,364 (34.1)	116 (3.6)
Yes	1,557 (15.4)	6,504 (65.9)	3,149 (96.5)
**Radiotherapy**			
No	3,208 (31.8)	4,501 (45.6)	436 (13.4)
Yes	6,893 (68.2)	5,367 (54.4)	2,829 (86.7)
**Adjuvant systemic therapy**			
No	8,012 (79.3)	1,808 (18.3)	532 (16.3)
Endocrine therapy	883 (8.7)	3,440 (34.9)	851 (26.1)
Chemotherapy	564 (5.6)	1,416 (14.4)	562 (17.2)
Both	642 (6.4)	3,204 (32.5)	1,320 (40.4)
**Primary systemic therapy**			
No	10,055 (99.5)	9,322 (94.5)	2,659 (81.4)
Yes	46 (0.5)	546 (5.5)	606 (18.6)
**Targeted therapy**			
No	9,853 (97.5)	8,936 (90.6)	2,756 (84.4)
Yes	248 (2.5)	932 (9.4)	509 (15.6)

Numbers are n (%). Abbreviations: ER = estrogen receptor, PR = progesterone receptor, HER2 = human epidermal growth factor receptor.

### Prediction models

For all underlying models estimating the mortality risk for stage I, we selected the following predictive variables: age, location within the breast, tumor differentiation grade, histological tumor type, multifocality, hormonal receptor status (ER/PR status), HER2 status, type of surgery, axillary lymph node dissection, use of adjuvant systemic therapy, primary systemic therapy, targeted therapy and radiotherapy. For the underlying models estimating the mortality risk for stage II, we selected the same predictive variables as for stage I, but added the variables tumor stage and nodal stage. For the underlying models predicting the mortality risk for stage III, the same predictive variables as for stage II were used. All 30 underlying models, including the 95% CIs, were embedded in a prediction model which is available on https://predictiemodel.verzekeraars.nl. A screenshot of the prediction model, currently available in Dutch, is shown in S1 Fig.

### Validation

All goodness-of-fit tests for all underlying models were non-significant, indicating no significant difference between observed and predicted mortality (Table 2). Discrimatory accuracy was moderate to good for all models with AUCs ranging from 0.69–0.90 (Table 2). After bootstrapping, the bias-corrected AUCs ranged from 0.67–0.88 (Table 2), indicating moderate to good discriminatory accuracy for all underlying models as a measure for internal validation.

**Table 2:** Overview of the Hosmer-Lemeshow test, the AUC and the bias-corrected AUC as measures for goodness-of-fit, discriminatory accuracy and internal validation of the models.

	Stage I (n = 10,101)			Stage II (n = 9,868)			Stage III (n = 3,265)		
Model	HL test p-value	AUC	Bias-corrected AUC	HL test p-value	AUC	Bias-corrected AUC	HL test p-value	AUC	Bias-corrected AUC
**Year 0–1**	0.499	0.81 (0.76–0.86)	0.79 (0.73–0.89)	0.087	0.86 (0.84–0.89)	0.85 (0.76–0.90)	0.351	0.90 (0.88–0.93)	0.89 (0.77–0.94)
**Year 1–2**	0.896	0.76 (0.72–0.79)	0.75 (0.69–0.81)	0.190	0.80 (0.78–0.82)	0.79 (0.75–0.83)	0.539	0.83 (0.80–0.85)	0.81 (0.77–0.86)
**Year 2–3**	0.883	0.74 (0.71–0.76)	0.73 (0.67–0.79)	0.577	0.75 (0.73–0.77)	0.74 (0.70–0.78)	0.089	0.77 (0.75–0.79)	0.75 (0.70–0.80)
**Year 3–4**	0.251	0.72 (0.69–0.74)	0.70 (0.65–0.76)	0.601	0.75 (0.74–0.76)	0.74 (0.70–0.79)	0.385	0.74 (0.72–0.76)	0.72 (0.65–0.78)
**Year 4–5**	0.679	0.71 (0.68–0.73)	0.70 (0.65–0.75)	0.291	0.71 (0.69–0.72)	0.70 (0.65–0.75)	0.190	0.74 (0.72–0.76_	0.71 (0.66–0.78)
**Year 5–6**	0.229	0.70 (0.67–0.72)	0.68 (0.63–0.74)	0.944	0.73 (0.72–0.75)	0.72 (0.67–0.77)	0.713	0.73 (0.72–0.74)	0.69 (0.60–0.77)
**Year 6–7**	0.579	0.72 (0.70–0.75)	0.70 (0.65–0.76)	0.644	0.73 (0.72–0.74)	0.72 (0.66–0.77)	0.286	0.71 (0.69–0.73)	0.67 (0.58–0.74)
**Year 7–8**	0.746	0.68 (0.66–0.70)	0.67 (0.62–0.72)	0.513	0.71 (0.70–0.73)	0.70 (0.64–0.78)	0.203	0.74 (0.72–0.76)	0.70 (0.64–0.78)
**Year 8–9**	0.291	0.74 (0.72–0.76)	0.73 (0.68–0.78)	0.595	0.74 (0.73–0.75)	0.73 (0.68–0.78)	0.231	0.73 (0.71–0.75)	0.69 (0.60–0.76)
**Year 9–10**	0.632	0.71 (0.69–0.72)	0.70 (0.65–0.76)	0.572	0.77 (0.76–0.79)	0.76 (0.71–0.81)	0.178	0.72 (0.70–0.74)	0.68 (0.59–0.78)

Abbreviations: HL = Hosmer-Lemeshow; AUC = Area under the receiver operating characteristic curve.

For external validation on patients diagnosed in 2007–2008, 24,761 patients were included of who 11,227 (45.3%) had stage I, 10,410 (42.0%) stage II and 3,124 (12.6%) stage III. Baseline characteristics of this validation population are shown in S1 Table. The external validation showed good calibration for all models. Calibration was expressed as the expected percentage of mortality using the newly-developed model minus the observed percentage of mortality in the validation population. Although several models showed a statistically significant difference between expected and observed mortality, the percentages were very small (<2% for 1 model, <1% for all other models). Discriminatory accuracy was moderate to good for all models with AUCs ranging from 0.61–0.87 (Table 3). As we know that breast cancer subtypes play an important role in breast cancer prognosis, we additionally performed the validation for ER positive, HER2 positive and triple negative breast cancer patients. Calibration of the model was satisfying in all three groups, with differences between expected and observed mortality under 3% (S2–S4 Tables). Discriminatory accuracy was moderate to good for ER positive breast cancer patients (AUCs ranging from 0.63 to 0.84) (S2 Table). For HER2 positive and triple negative patients, the AUCs for most of the models were moderate to good, but several models showed poor discriminatory accuracy (with wide confidence intervals), caused by the low numbers of events in these years (S3 and S4 Tables).

**Table 3:** Calibration and discrimination of the model on the external validation population (2007–2008, n = 24,761).

	Stage I (n = 11,227)		Stage II (n = 10,410)		Stage III (n = 3,124)	
Model	Expected–observed (95% CI)	AUC	Expected–observed (95% CI)	AUC	Expected–observed (95% CI)	AUC
**Year 0–1**	0.28 (0.26–0.30)	0.71 (0.65–0.78)	-0.10 (-0.12 to -0.08)	0.87 (0.75–0.82)	-0.80 (-0.84 to -0.76)	0.86 (0.83–0.90)
**Year 1–2**	-0.66 (-0.68 to -0.64)	0.68 (0.64–0.72)	0.31 (0.29–0.34)	0.76 (0.73–0.79)	-0.26 (-0.30 to -0.22)	0.81 (0.77–0.84)
**Year 2–3**	-0.11 (-0.13 to -0.09)	0.66 (0.62–0.70)	0.30 (0.28–0.32)	0.72 (0.69–0.75)	-0.21 (-0.25 to -0.17)	0.74 (0.70–0.78)
**Year 3–4**	0.44 (0.42–0.46)	0.65 (0.60–0.69)	-0.28 (-0.30 to -0.26)	0.69 (0.66–0.72)	-0.40 (-0.44 to -0.36)	0.69 (0.65–0.73)
**Year 4–5**	-0.73 (-0.75 to -0.71)	0.63 (0.59–0.67)	-0.17 (-0.19 to -0.15)	0.70 (0.67–0.73)	0.19 (0.15–0.24)	0.61 (0.56–0.66)
**Year 5–6**	-0.02 (-0.04 to 0.00)	0.67 (0.64–0.71)	0.53 (0.51–0.55)	0.70 (0.67–0.73)	-0.06 (-0.10 to -0.01)	0.66 (0.60–0.71)
**Year 6–7**	0.14 (0.12–0.16)	0.69 (0.65–0.72)	0.07 (0.05–0.09)	0.69 (0.66–0.73)	1.51 (1.46–1.56)	0.68 (0.62–0.74)
**Year 7–8**	-0.11 (-0.13 to -0.09)	0.66 (0.63–0.70)	0.46 (0.44–0.49)	0.70 (0.66–0.74)	0.94 (0.89–0.98)	0.64 (0.58–0.74)
**Year 8–9**	-	-	-	-	-	-
**Year 9–10**	-	-	-	-	-	-

Calibration is expressed as the expected mortality of the newly-developed model minus the observed mortality, both in percentages, in the validation population. Validation of the last two models per stage was not possible due to lack of follow-up after eight years. Abbreviations: AUC = area under the receiver operating characteristic curve.

### Model testing

**Fig 1:**
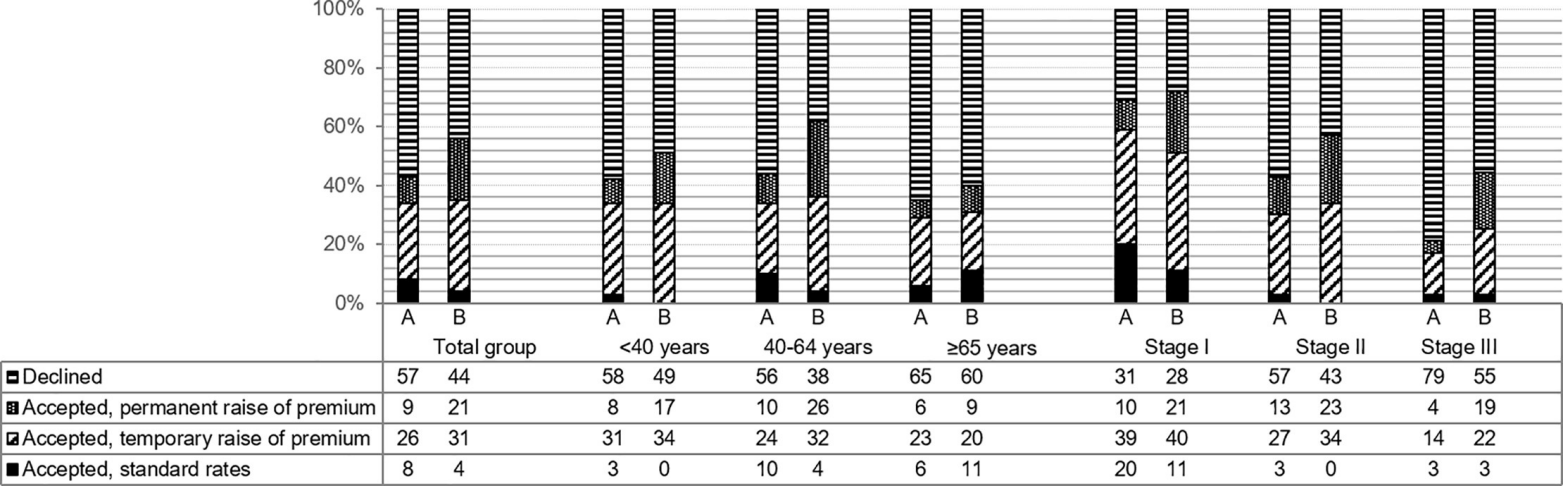
Comparison of insurability of (ex-)breast cancer patients between currently used guidelines (A) and the newly-developed model (B).

Altogether, eight of the 10 life insurers responded. Fig 1 summarizes the insurability of the 50 cases for every stage and age category. Overall, the insurability increased from 43%-56%. However, the lower number of declined applications resulted in a higher number of accepted applications with temporary or permanent premium raises. The number of applications accepted at standard rates decreased from 8%-4%. Patients aged 40–64 and patients with stage III breast cancer benefited the most of the newly-developed model compared to the currently used guidelines: insurability (with or without premium raise) increased from 44%-62% and from 21%-45%, respectively.

**Fig 2:**
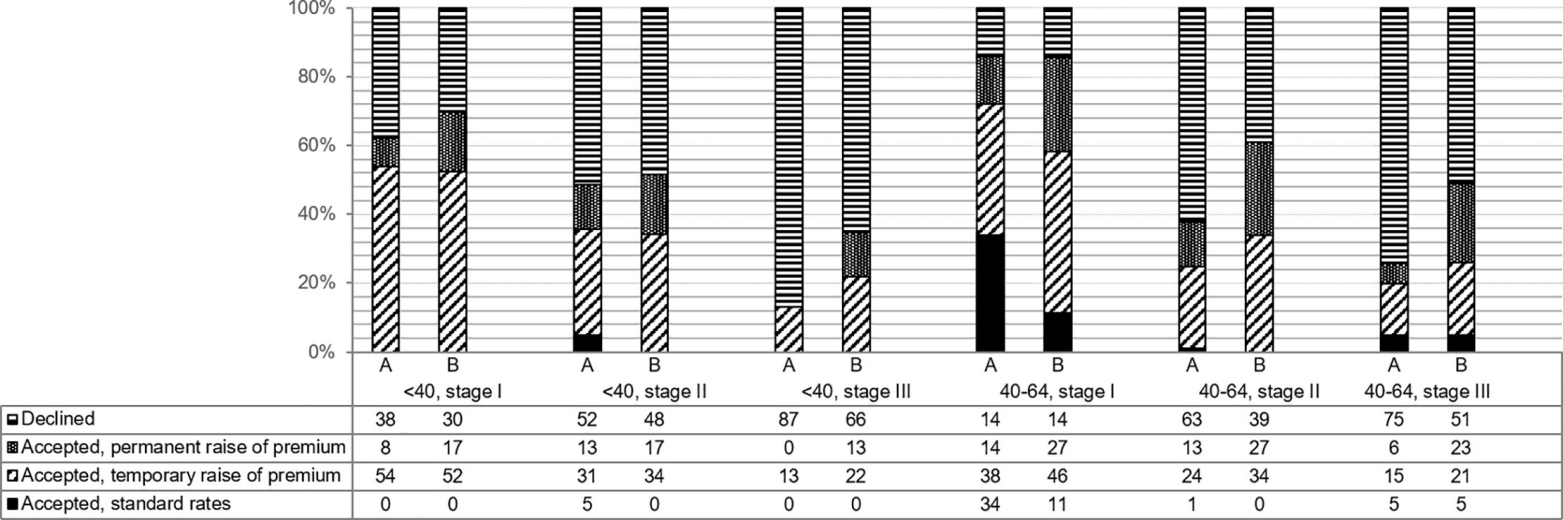
Further specification of the comparison of insurability of (ex-)breast cancer patients between current guidelines (A) and the newly-developed model (B).

Results were further specified for the age groups <40 and 40–64 years (for the group ≥65 years numbers were too small) (Fig 2). For all (ex-)patients<40 years, the percentage of rejections decreased by using the newly-developed model. As more patients were accepted, temporary or permanent premium increases were more common. For patients<40 years with stage II disease, the number of acceptations at standard rates decreased from 5%-0%. (Ex-)patients age <40 with stage III breast cancer benefit the most from the new model, with acceptance rates increasing from 13% using the current guidelines to 34% with the newly-developed model. (Ex-)patients aged 40–64 with stage I disease showed the least benefit of the newly-developed model. The number of rejections remained identical, but the number of acceptations at standard rates declined from 34%-11% using the new model. However, acceptance rates were for this group the highest, namely 86%. For patients aged 40–64 with stage II and III disease, the newly-developed model showed less rejections, and the number of acceptations at standard rates remained similar.

## Discussion

A conditional prediction model for the annual extra mortality risk of breast cancer patients compared to the general population was generated. This prediction model provides life insurers with more reliable information to base their decision on, which is shown to result in increased insurability of breast cancer patients in a test using 50 random patients.

The benefit of the newly-developed model in terms of insurability was greatest for patients with stage III breast cancer. Especially for (ex-)patients<40 years with stage III disease, insurability was almost tripled. This is of great importance as this group mostly applies for a life insurance, as part of a mortgage. For patients with stage I disease, the newly-developed model did not add much benefit in terms of insurability. This may be explained by the fact that higher staged breast cancers, when recurring, most often recur in the first few years following diagnosis[22]. Being alive an increasing number of years following diagnosis considerably lowers the remaining risk on locoregional recurrences and distant metastases and consequently the risk of death.

As this model is specifically designed for the number of years survived, it provides life insurers enhanced insight in increasing survival probabilities over time, resulting in a higher chance to get accepted. The higher acceptance rates were mainly acceptances with premium increases, as patients whose applications were first rejected are now mostly accepted against a raised premium. The exact premium raise could not be specified due to life insurers’ competition policy, the Antitrust legislation, and the relatively low number of tested cases. However, these premium raises varied widely between life insurers. Some patients were first accepted at standard rates, but had to pay a premium raise using the newly-developed model. Fortunately, these raises were low and may not outweigh the increased number of acceptations. Furthermore, an application of a specific patient which is rejected by one life insurer may be accepted by another. Although our model provides a more objective basis for life insurers to base their decision on, it does not change the large differences in the consequences for the premium. We showed that for patients aged 40–64 with stage I disease, the number of acceptations at standard rates decreased from 34%-11% using the new model. Notably, every life insurer tested the model on the same 50 cases, so results are presented at the level of the life insurers, and not on individual level. A sensitivity analysis of the data showed that, when individual patients would apply at multiple life insurers, every patient in this test was insurable (either at standard rates or with premium raises). This information should, however, be cautiously interpreted, as premiums are sometimes very high. Therefore, breast cancer patients and survivors–especially patients aged 40–64 with stage I disease–are advised to apply for a life insurance at different life insurers, and to compare the outcomes.

Multiple prediction models for breast cancer have been developed, of which many predict the overall mortality risk[23–27]. One of these models, the Breast Cancer Conditional Outcome Calculator (CancerMath.net)[26], estimates conditional survival, thereby increasing its usability for patients who survived several years. Accuracy of this model was considered to be modest in Southeast Asian women with breast cancer[28]. This model, however, does not provide the annual mortality risks and does not show uncertainty around the estimates. Especially the latter is important, since communication of risk prediction models to patients is shown to be very difficult[29].

Our study has several strengths. First, we corrected patients’ mortality rates for the mortality rates of the general Dutch population. Herewith, more information on a patient’s individual extra risk is provided. Second, we used the NCR to cover the entire Dutch breast cancer population, thereby enhancing the generalizability of the model. Third, a large number of patients (n = 23,234) was included, allowing us to include a large number of predictive factors. The NCR has a completeness of over 95% and is considered to be of high quality due to very tumor-specific guidelines, trained data managers and regular quality controls. This data is therefore representative for the entire non-metastatic breast cancer population. A limitation is the lack of knowledge on performance status or comorbidities. Irrespective of a previous cancer diagnosis, comorbidity may lead to a lower chance to be insured. Furthermore, cancer patients with comorbidities are less likely to receive the standard cancer treatments, and their risk of postoperative complications is higher compared to patients without comorbidities, resulting in lower overall survival rates[30–32]. However, by adjusting the observed mortality for the expected mortality risks of the general population, matched by age, gender and calendar year, we partly solved this problem by correction for age- and gender-related comorbidities. Furthermore, we lacked data on recurrences during follow-up. This might lead to an overestimated mortality risk for patients who are free of disease during follow-up and an underestimated mortality risk for patients diagnosed with a recurrence during follow-up. Another limitation is the use of the general Dutch population as a reference. Baseline mortality risks of other countries may differ from those in the Netherlands, which may have consequences for the applicability of this model in other countries. Therefore, before using this model on a specific target population, the model should be validated on that population. Predictive modelling in general has limitations, as the risk produced by a certain model is based on an underlying model population with certain characteristics that are most often collected retrospectively. These patients have been treated according to clinical guidelines that were valid at time of diagnosis, which is in this case over 10 years ago. One can imagine that results of this specific underlying population may be different in future patients. Besides, since two patients with exactly similar characteristics may respond completely different on a certain treatment, it should be communicated to patients that the risk produced by the model does not have to count for them per se. Therefore, it is vital to take any other prognostic information into account, such as development of new therapies and outcomes of randomized controlled trials. Lastly, life insurances often last for 30 years. Our model only includes information until 10 years from diagnosis. As a very important prognostic factor–the HER2 status–was introduced in 2005, and patients were also treated accordingly, it was decided not to include patients diagnosed before 2005. The fact we included the more contemporary treatment regimens and important knowledge on receptor statuses outweighs in our opinion the limited follow-up time. Lastly, several aspects on data interpretation have to be discussed. Although a large number of patients is included, outcomes of very specific combinations of variables in a specific year following diagnosis may still be hard to interpret, as they may sometimes reflect a low number of cases and events. For this reason, it is of crucial importance to evaluate the confidence intervals around the estimates. If these are very wide, one should be careful in interpreting the results, as a certain outcome may be due to chance. Results of this model are not meant to directly be translated into raises of premium, but they are there to support life insurers in their decision. Any other information, such as the use of new therapies, presence of comorbidities or recent literature should be taken into account.

The Dutch Association of Insurers stimulates the use of the model by life insurers by providing information on their website and in several meetings. The model is currently being used by several Dutch life insurers in practice, who are documenting both the results of the new model and that of their current guidelines for a year, for every (ex-)breast cancer patient who applies for a life insurance. After a year, these results will be evaluated and consequently used for improving the model or identification of any remaining problems.

The results of our study are not directly applicable to other countries, as we specifically generated this model for patients diagnosed with breast cancer in the Netherlands (including the use of a Dutch reference population), to optimize the decision-making process in the application for a life insurance. The model can, of course, be validated on other populations to determine its accuracy. However, with this model we hope to active other researchers in other countries to generate a similar model for their own population. As many (ex-)cancer patients from many countries are still experiencing difficulties in getting a life insurance, it is of crucial importance to use representative and up to date information on extra mortality risks. Creating a prediction model on the same population as were it is applied on results in the most accurate predictions.

## Conclusions

In this study, a conditional prediction model for the annual extra mortality risk of breast cancer patients compared to the general population was generated. This model was internally and externally validated and was tested in practice with satisfying results. The model is publicly available and ready to be used by life insurers in the Netherlands. By providing all life insurers the same base for their decision, we hope to increase transparency to patients. For a year, all applications by breast cancer patients and survivors will be processed by life insurers using both the current guidelines and the newly-developed model. Thereafter, the results will be analyzed and included in further discussions on insurability of breast cancer patients and survivors between the Dutch Federation of Cancer Patient Organizations and the Dutch Association of Insurers. Furthermore, the model will be updated regularly, to ensure that life insurers work with the most recent and reliable data. In future, prediction models for other types of cancers will be developed.

## Supporting information

S1 Table Patient-, tumour-, and treatment-related characteristics of the validation population (2007–2008). (DOCX)

S2 Table Calibration and discrimination of the model on the external validation population for ER positive patients (2007–2008, n = 19,968). (DOCX)

S3 Table Calibration and discrimination of the model on the external validation population for HER2 positive patients (2007–2008, n = 3,249). (DOCX)

S4 Table Calibration and discrimination of the model on the external validation population for triple negative patients (2007–2008, n = 2,834). (DOCX)

S1 Fig Screenshot of prediction model using data of a fictive patient, in Dutch. (DOCX)
