# Supplementary material for: A conditional model predicting the 10-year annual extra mortality risk compared to the general population: a large population-based study in Dutch breast cancer patients
Source: PLoS One. 2019 Jan 24;14(1):e0210887. doi: 10.1371/journal.pone.0210887 (PMC6345454; doi:10.1371/journal.pone.0210887)
Supplement: S1 Table — (DOCX) [file pone.0210887.s001.docx]

**S1 Table. Patient-, tumour-, and treatment-related characteristics of the validation population (2007-2008)**

| **Characteristics** | **Stage I (n=11,227)** | **Stage II (n=10,410)** | **Stage III (n=3,124)** |
| --- | --- | --- | --- |
| **Age (years)** |  |  |  |
| <40 | 443 (4.0) | 648 (6.2) | 251 (8.0) |
| 40-64 | 6,476 (57.7) | 6,093 (58.5) | 1,884 (60.3) |
| ≥65 | 4,308 (38.4) | 3,669 (35.2) | 989 (31.7) |
| **Pathological tumour stage** |  |  |  |
| T1 | 11,186 (99.6) | 3,147 (30.2) | 650 (20.8) |
| T2 | - | 6,936 (66.6) | 1,326 (42.5) |
| T3 | - | 281 (2.7) | 646 (20.7) |
| T4 | - | - | 470 (15.0) |
| Unknown | 41 (0.4) | 46 (0.4) | 32 (1.0) |
| **Pathological nodal stage** |  |  |  |
| N0 | 10,967 (97.7) | 4,201 (40.4) | 122 (3.9) |
| N1 | - | 6,058 (58.2) | 630 (20.2) |
| N2 | - | - | 1,468 (47.0) |
| N3 | - | - | 872 (27.9) |
| Unknown | 260 (2.3) | 151 (1.5) | 32 (1.0) |
| **Lateralisation** |  |  |  |
| Left | 5,749 (51.2) | 5,326 (51.2) | 1,649 (52.8) |
| Right | 5,477 (48.8) | 5,083 (48.8) | 1,475 (47.2) |
| Unknown | 1 (0.0) | 1 (0.0) | - |
| **Sublocation** |  |  |  |
| Outer quadrants | 5,150 (45.9) | 4,908 (47.2) | 1,385 (44.3) |
| Inner quadrants | 2,564 (22.8) | 1,783 (17.1) | 340 (10.9) |
| Central parts | 709 (6.3) | 868 (8.3) | 273 (8.7) |
| Overlapping lesions | 2,582 (23.0) | 2,675 (25.7) | 1,050 (33.6) |
| Unknown | 222 (2.0) | 176 (1.7) | 76 (2.4) |
| **Differentiation grade** |  |  |  |
| 1 | 3,577 (31.9) | 1,651 (15.9) | 260 (8.3) |
| 2 | 4,775 (42.5) | 4,287 (41.2) | 1,097 (35.1) |
| 3 | 2,325 (20.7) | 3,611 (34.7) | 1,260 (40.3) |
| Unknown | 550 (4.9) | 861 (8.3) | 507 (16.2) |
| **Histological tumour type** |  |  |  |
| Ductal | 9,248 (82.4) | 8,211 (78.9) | 2,427 (77.7) |
| Lobular | 991 (8.8) | 1,257 (12.1) | 489 (15.7) |
| Mixed | 421 (3.8) | 474 (4.6) | 143 (4.6) |
| Other | 567 (5.1) | 468 (4.5) | 65 (2.1) |
| **Multifocality** |  |  |  |
| No | 9,638 (85.9) | 8,459 (81.3) | 2,214 (70.9) |
| Yes | 1,512 (13.5) | 1,836 (17.6) | 829 (26.5) |
| Unknown | 77 (0.7) | 115 (1.1) | 81 (2.6) |
| **Hormonal receptor status** |  |  |  |
| ER and PR positive | 7,648 (68.1) | 6,625 (63.6) | 1,761 (56.4) |
| ER or PR positive | 1,914 (17.1) | 1,737 (16.7) | 595 (19.1) |
| ER negative | 1,481 (13.2) | 1,988 (19.1) | 758 (24.3) |
| Unknown | 184 (1.6) | 60 (0.6) | 10 (0.3) |
| **HER2 status** |  |  |  |
| Negative | 9,428 (84.0) | 8,647 (83.1) | 2,317 (74.2) |
| Unclear | 339 (3.0) | 87 (0.8) | 18 (0.6) |
| Positive | 1,113 (9.9) | 1,413 (13.6) | 723 (23.1) |
| Unknown | 347 (3.1) | 263 (2.5) | 66 (2.1) |
| **Type of surgery** |  |  |  |
| Breast-conserving surgery | 7,695 (68.5) | 4,854 (46.6) | 713 (22.8) |
| Mastectomy | 3,521 (31.4) | 5,554 (53.4) | 2,411 (77.2) |
| Unknown | 11 (0.1) | 2 (0.0) | - |
| **Axillary lymph node dissection** |  |  |  |
| No | 10,126 (90.2) | 3,959 (38.0) | 109 (3.5) |
| Yes | 1,101 (9.8) | 6,451 (62.0) | 3,015 (96.5) |
| **Radiation therapy** |  |  |  |
| No | 3,611 (32.2) | 4,627 (44.5) | 423 (13.5) |
| Yes | 7,616 (67.8) | 5,783 (55.6) | 2,701 (86.5) |
| **Adjuvant systemic therapy** |  |  |  |
| No | 8,202 (73.1) | 1,766 (17.0) | 513 (16.4) |
| Endocrine therapy | 1,432 (12.8) | 3,773 (36.2) | 819 (26.2) |
| Chemotherapy | 750 (6.7) | 1,545 (14.8) | 524 (16.8) |
| Both | 843 (7.5) | 3,326 (32.0) | 1,268 (40.6) |
| **Primary systemic therapy** |  |  |  |
| No | 11,133 (99.2) | 9,469 (90.7) | 2,471 (79.1) |
| Yes | 94 (0.8) | 941 (9.0) | 653 (20.9) |
| **Targeted therapy** |  |  |  |
| No | 10,873 (96.9) | 9,379 (90.1) | 2,519 (80.6) |
| Yes | 354 (3.2) | 1,031 (9.9) | 605 (19.4) |

Numbers are n (%). Abbreviations: ER = estrogen receptor, PR = progesterone receptor, HER2 = human epidermal growth factor receptor.
