# Supplementary material for: A conditional model predicting the 10-year annual extra mortality risk compared to the general population: a large population-based study in Dutch breast cancer patients
Source: PLoS One. 2019 Jan 24;14(1):e0210887. doi: 10.1371/journal.pone.0210887 (PMC6345454; doi:10.1371/journal.pone.0210887)
Supplement: S2 Table — (DOCX) [file pone.0210887.s002.docx]

**S2 Table. Calibration and discrimination of the model on the external validation population for ER positive patients (2007-2008, n=19,968)**

|  | **Stage I (n=9,455)** | | **Stage II (n=8,212)** | | **Stage III (n=2,301)** | | |
| --- | --- | --- | --- | --- | --- | --- | --- |
| **Model** | **Expected – observed (95% CI)** | **AUC** | **Expected – observed (95% CI)** | **AUC** | **Expected – observed (95% CI)** | **AUC** |  |
| **Year 0-1** | 0.37 (0.37-0.37) | 0.73 (0.66-0.80) | 0.07 (0.07-0.07) | 0.77 (0.73-0.82) | -0.53 (-0.53 to -0.52) | 0.74 (0.78-0.90) |  |
| **Year 1-2** | -0.67 (-0.68 to -0.67) | 0.69 (0.64-0.74) | 0.10 (0.10-0.11) | 0.76 (0.72-0.79) | 0.38 (0.38-0.39) | 0.76 (0.70-0.82) |  |
| **Year 2-3** | -0.15 (-0.14 to -0.15) | 0.66 (0.61-0.67) | 0.14 (0.13-0.14) | 0.72 (0.68-0.75) | -0.24 (-0.25 to -0.23) | 0.71 (0.65-0.76) |  |
| **Year 3-4** | 0.46 (0.46-0.46) | 0.63 (0.58-0.68) | -0.51 (-0.51 to -0.50) | 0.69 (0.66-0.73) | -0.30 (-0.32 to -0.29) | 0.68 (0.63-0.73) |  |
| **Year 4-5** | -0.70 (-0.71 to 0.70) | 0.64 (0.60-0.68) | -0.47 (-0.48 to -0.47) | 0.71 (0.68-0.75) | 0.09 (0.08-0.10) | 0.63 (0.57-0.69) |  |
| **Year 5-6** | 0.07 (0.07-0.07) | 0.67 (0.63-0.71) | 0.50 (0.49-0.50) | 0.69 (0.66-0.73) | -0.23 (-0.24 to -0.22) | 0.68 (0.63-0.74) |  |
| **Year 6-7** | 0.03 (0.03-0.03) | 0.69 (0.65-0.73) | 0.28 (0.28-0.28) | 0.70 (0.66-0.74) | 1.70 (1.69-1.72) | 0.68 (0.62-0.74) |  |
| **Year 7-8** | -0.12 (-0.13 to -0.12) | 0.66 (0.62-0.70) | 0.50 (0.50-0.50) | 0.71 (0.67-0.75) | 0.79 (0.78-0.80) | 0.64 (0.58-0.71) |  |
| **Year 8-9** | - | - | - | - | - | - |  |
| **Year 9- 10** | - | - | - | - | - | - |  |

Calibration is expressed as the expected mortality of the newly-developed model minus the observed mortality, both in percentages, in the validation population. Validation of the last two models per stage was not possible due to lack of follow-up after eight years. Abbreviations: AUC = area under the receiver operating characteristic curve.
