# Supplementary material for: A conditional model predicting the 10-year annual extra mortality risk compared to the general population: a large population-based study in Dutch breast cancer patients
Source: PLoS One. 2019 Jan 24;14(1):e0210887. doi: 10.1371/journal.pone.0210887 (PMC6345454; doi:10.1371/journal.pone.0210887)
Supplement: S3 Table — (DOCX) [file pone.0210887.s003.docx]

**S3 Table. Calibration and discrimination of the model on the external validation population for HER2 positive patients (2007-2008, n=3,249)**

|  | **Stage I (n=1,113)** | | **Stage II (n=1,413)** | | | **Stage III (n=723)** | | |
| --- | --- | --- | --- | --- | --- | --- | --- | --- |
| **Model** | **Expected – observed (95% CI)** | **AUC** | | **Expected – observed (95% CI)** | **AUC** | | **Expected – observed (95% CI)** | **AUC** |
| **Year 0-1** | -0.16 (-0.17 to -0.16) | 0.75 (0.55-0.94) | | -0.41 (-0.41 to -0.40) | 0.79 (0.71-0.88) | | -0.73 (-0.74 to -0.71) | 0.88 (0.77-0.98) |
| **Year 1-2** | -0.92 (-0.93 to -0.92) | 0.65 (0.50-0.80) | | -0.70 (-0.71 to -0.69) | 0.77 (0.70-0.85) | | -0.70 (-0.72 to -0.67) | 0.84 (0.76-0.91) |
| **Year 2-3** | 0.15 (0.14-0.16) | 0.70 (0.57-0.83) | | 0.23 (0.22-0.24) | 0.71 (0.61-0.82) | | -0.28 (-0.31 to -0.25) | 0.73 (0.63-0.82) |
| **Year 3-4** | 1.68 (1.67-1.68) | 0.57 (0.38-0.77) | | -0.04 (-0.05 to -0.03) | 0.60 (0.51-0.69) | | -0.45 (-0.47 to -0.42) | 0.68 (0.57-0.79) |
| **Year 4-5** | 0.35 (0.34-0.35) | 0.63 (0.40-0.86) | | 0.37 (0.36-0.38) | 0.77 (0.69-0.85) | | 2.09 (2.07-2.11) | 0.54 (0.43-0.65) |
| **Year 5-6** | 0.02 (0.01-0.03) | 0.67 (0.56-0.78) | | 0.89 (0.88-0.90) | 0.71 (0.61-0.81) | | -0.36 (-0.39 to -0.32) | 0.56 (0.44-0.68) |
| **Year 6-7** | 0.46 (0.45-0.46) | 0.76 (0.62-0.89) | | -0.93 (-0.94 to -0.92) | 0.64 (0.51-0.76) | | 2.19 (2.17-2.20) | 0.65 (0.44-0.85) |
| **Year 7-8** | 1.24 (1.23-1.25) | 0.57 (0.46-0.68) | | -0.98 (-0.99 to -0.97) | 0.56 (0.43-0.70) | | 1.49 (1.47-1.51) | 0.48 (0.26-0.71) |
| **Year 8-9** | - | - | | - | - | | - | - |
| **Year 9- 10** | - | - | | - | - | | - | - |

Calibration is expressed as the expected mortality of the newly-developed model minus the observed mortality, both in percentages, in the validation population. Validation of the last two models per stage was not possible due to lack of follow-up after eight years. Abbreviations: AUC = area under the receiver operating characteristic curve.
