# Supplementary material for: A conditional model predicting the 10-year annual extra mortality risk compared to the general population: a large population-based study in Dutch breast cancer patients
Source: PLoS One. 2019 Jan 24;14(1):e0210887. doi: 10.1371/journal.pone.0210887 (PMC6345454; doi:10.1371/journal.pone.0210887)
Supplement: S4 Table — (DOCX) [file pone.0210887.s004.docx]

**S4 Table. Calibration and discrimination of the model on the external validation population for triple negative patients (2007-2008, n=2,834)**

|  | **Stage I (n=1,051)** | | **Stage II (n=1,382)** | | **Stage III (n=401)** | |
| --- | --- | --- | --- | --- | --- | --- |
| **Model** | **Expected – observed (95% CI)** | **AUC** | **Expected – observed (95% CI)** | **AUC** | **Expected – observed (95% CI)** | **AUC** |
| **Year 0-1** | -0.11 (-0.12 to -0.11) | 0.69 (0.48-0.89) | -1.09 (-1.11 to -1.08) | 0.75 (0.68-0.83) | -2.95 (-2.99 to -2.92) | 0.76 (0.67-0.84) |
| **Year 1-2** | -0.01 (-0.02 to 0.00) | 0.62 (0.50-0.75) | 1.66 (1.65 to 1.68) | 0.75 (0.68-0.83) | -2.95 (-3.00 to -2.90) | 0.60 (0.51-0.68) |
| **Year 2-3** | -0.06 (-0.07 to -0.05) | 0.59 (0.47-0.70) | 1.21 (1.20-1.22) | 0.61 (0.55-0.69) | -2.12 (-2.17 to -2.1) | 0.69 (0.60-0.79) |
| **Year 3-4** | -0.25 (-0.26 to -0.24) | 0.67 (0.56-0.78) | 0.97 (0.96-0.99) | 0.68 (0.60-0.75) | -1.91 (-1.96 to -1.86) | 0.63 (0.52-0.75) |
| **Year 4-5** | -1.53 (-1.54 to -1.51) | 0.53 (0.40-0.66) | 1.10 (1.09-1.11) | 0.58 (0.49-0.68) | -3.42 (-3.45 to -3.37) | 0.48 (0.34-0.62) |
| **Year 5-6** | -0.98 (-0.99 to -0.96) | 0.65 (0.56-0.75) | 0.57 (0.56-0.58) | 0.71 (0.62-0.81) | -0.14 (-0.18 to -0.11) | 0.41 (0.20-0.62) |
| **Year 6-7** | 0.94 (0.93-0.95) | 0.60 (0.45-0.74) | -0.59 (-0.60 to -0.58) | 0.68 (0.55-0.81) | -0.46 (-0.50 to -0.42) | 0.60 (0.36-0.85) |
| **Year 7-8** | -0.24 (-0.25 to -0.23) | 0.67 (0.53-0.81) | 0.80 (0.79-0.81) | 0.73 (0.61-0.86) | 1.79 (1.75-1.83) | 0.64 (0.40-0.88) |
| **Year 8-9** | - | - | - | - | - | - |
| **Year 9- 10** | - | - | - | - | - | - |

Calibration is expressed as the expected mortality of the newly-developed model minus the observed mortality, both in percentages, in the validation population. Validation of the last two models per stage was not possible due to lack of follow-up after eight years. Abbreviations: AUC = area under the receiver operating characteristic curve.
