# Supplementary material for: A conditional model predicting the 10-year annual extra mortality risk compared to the general population: a large population-based study in Dutch breast cancer patients
Source: PLoS One. 2019 Jan 24;14(1):e0210887. doi: 10.1371/journal.pone.0210887 (PMC6345454; doi:10.1371/journal.pone.0210887)
Supplement: S1 Fig — (DOCX) [file pone.0210887.s005.docx]

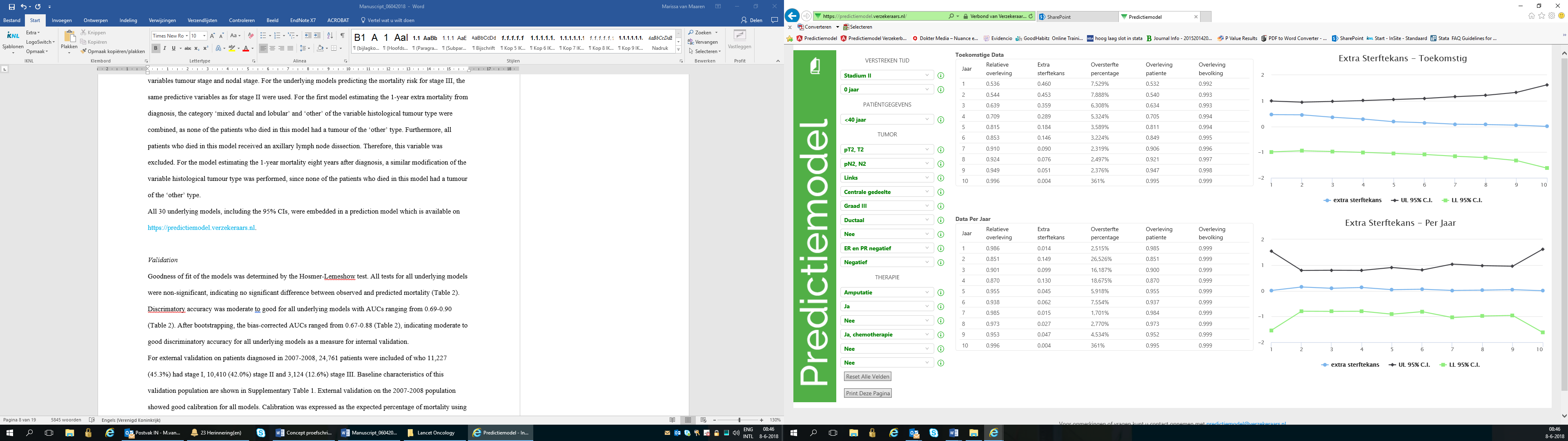


**S1 Fig. Screenshot of prediction model using data of a fictive patient, in Dutch.**

Reprinted from http://www.predictiemodel.verzekeraars.nl/ under a CC BY license, with permission from The Dutch Association of Insurers, original copyright [2017].
